# Pan‐Cancer analyses of Necroptosis‐Related genes as a potential target to predict immunotherapeutic outcome

**DOI:** 10.1111/jcmm.17634

**Published:** 2022-12-29

**Authors:** Zheng Zhou, Jiajun Wu, Wenli Ma, Feilin Dong, Jiafeng Wang

**Affiliations:** ^1^ Bengbu Medical College Graduate School Bengbu China; ^2^ Department of Head and Neck Surgery, Centre of Otolaryngology‐Head and Neck Surgery, Zhejiang Provincial People's Hospital People's Hospital of Hangzhou Medical College Hangzhou China; ^3^ Zhejiang Provincial Key Laboratory of Diagnosis and Treatment of Endocrine Gland Diseases Hangzhou China

**Keywords:** cancer, immunotherapy, mutation, necroptosis, tumour microenvironment

## Abstract

Necroptosis is a unique programmed death mechanism of necrotic cells. However, its role and specific mechanism in cancer remain unclear, and a systematic pan‐cancer analysis of necroptosis is yet to be conducted. Thus, we performed a specific pan‐cancer analysis using The Cancer Genome Atlas and Genotype‐Tissue Expression databases to analyse necroptosis expression in terms of cancer prognosis, DNA methylation status, tumour mutative burden, microsatellite instability, immune cell infiltration in different types of cancer and molecular mechanisms. For the first time, we explored the correlation between necroptosis and immunotherapy prognosis. Thus, our study provides a relatively comprehensive understanding of the carcinogenicity of necroptosis in different types of cancer. It is suggested that necroptosis can be used to evaluate the sensitivity of different patients to immunotherapy and may become a potential target for tumour immunotherapy.

## 1 INTRODUCTION

Apoptosis is generally considered to be the most common form of programmed cell death and is distinct from necrosis.^1^ However, necrotic cell death can be achieved through another programmed cell death mechanism known as necroptosis, which is similar to the apoptosis and cell necrosis morphology.^2^

In 2005, Alexei Degterev et al.^3^ first reported necroptosis and demonstrated that necroptosis participates in delayed cerebral ischemia injury in mice through a mechanism different from apoptosis. Interestingly, subsequent studies have found that tumour necrosis factor‐α (TNF‐α) and TNF related apoptosis‐inducing ligand, including tumour necrosis factors, can activate both necroptosis and conventional apoptosis.^2^ Necroptosis often occurs when caspase‐8 activity is inhibited or blocked by drugs^4^ and receptor‐interacting serine/threonine kinase 1 (*RIPK1*) and receptor‐interacting serine/threonine kinase 3 (*RIPK3*) form an intracellular complex called the necrosome, which rapidly permeates through the mixed lineage kinase domain‐like pseudokinase (MLKL) protein downstream of *RIPK3* to activate necrotic cell death.^5^, ^6^, ^7^, ^8^ Necroptosis has been shown to be inhibited by the necroptosis inhibitor necrostatin‐1, which specifically inhibits *RIPK1* activity.^9^

Necroptosis is considered an important cell death mechanism in the human body and is closely related to tumour occurrence and development. In contrast to traditional apoptosis, necroptosis has certain proinflammatory effects and promotes tumour progression.^10^ However, further studies on the correlation between necroptosis and tumours have shown that these characteristics are not absolute. McCormick et al.^11^ found that the expression of *RIPK1*, an important necroptosis factor, is downregulated in head and neck squamous cell carcinoma, enabling tumour cells to evade anoikis, thus enhancing tumour invasion and metastasis. However, in glioblastoma, *RIPK1* expression is upregulated and correlated with poor prognosis.^12^ Therefore, necroptosis often elicits favourable and unfavourable effects on tumours depending on tumour type.

Necroptosis may be associated with tumour immunity.^13^ Yatim et al.^14^ demonstrated that CD8+ T cell activation by necrotic cell‐induced immune responses can protect mice from tumour invasion through the release of various effector cytokines. However, in pancreatic carcinoma, necroptosis often results in the formation of an immunosuppressive tumour microenvironment that promotes tumorigenesis.^15^

The specific mechanism of necroptosis in tumours remains unclear, with opposite effects observed in different tumours. Therefore, the role of necroptosis in cancer must be analysed from the pan‐cancer perspective. In our study, we integrated relevant literature and used tumour‐related data from The Cancer Genome Atlas (TCGA) and Genotype‐Tissue Expression (GTEx) databases based on eight necroptosis‐related genes (NRGs) in the Gene Ontology database. We performed a pan‐cancer analysis of necroptosis, discussed the correlation between the expression of NRGs and the prognosis of 33 cancer types, and explored the possible molecular mechanism through pathway enrichment analysis. We also investigated the NRGs related to mutation, tumour mutative burden (TMB), microsatellite instability (MSI) and immunological association with tumours. Our study presents novel ideas for the immunotherapy of clinical cancer.

## 2 MATERIALS AND METHODS

### 2.1 Obtaining necroptosis‐related genes

The Molecular Signatures Database (MSigDB, https://www.gsea‐msigdb.org/), an online database for human genes and 8 gene sets have been constructed from the perspectives of location, function, metabolic pathway, target binding and so on, was used. The ‘C5 Ontology Gene set’ module in the MSigDB, where the submodule ‘Gene Ontology biological process’, including 7658 gene sets, was opened by turns and the necroptosis sets were retrieved. The gene set included eight related genes in the following order: *RIPK3*, *MLKL*, *Fas* cell surface death receptor (*FAS*), fas ligand (*FASLG*), toll‐like receptor 3 *(TLR3)*, tumour necrosis factor (*TNF*), *RIPK1* and fas associated via death domain (*FADD*). In addition to *RIPK1*, *RIPK3* and *MLKL* mentioned earlier, regarding *FAS*, the interaction of this receptor with its ligand allows the formation of a death‐inducing signalling complex that includes *FADD* and leads to apoptosis. And *FASLG* could induce apoptosis triggered by *FAS* binding. All eight NRGs were then used for correlation analysis

### 2.2 Data collection and processing

RNA sequence, somatic mutation and related clinical data were downloaded from TCGA (comprising 10,497 samples from 33 cancer types) by using UCSC Xena (https://Xena.UCSC.edu/), an online tool for exploring gene expression and clinical and phenotypic data. Gene expression data from 31 normal tissues were downloaded from the GTEx database (https://commonfund.nih.gov/GTEx). The Gene Expression Omnibus database (https://www.ncbi.nlm.nih.gov/) was used to obtain gene expression levels, and related clinical traits of GSE135222 (including 27 immuno‐treated patients with non‐small‐cell lung carcinoma) and GSE176307 (including 90 immuno‐treated patients with bladder carcinoma) were collected for all samples.

### 2.3 NRG differential expression and correlation analysis

The R package ‘Limma’ in R software (https://www.r‐project.org/, version 4.1.3) was used to analyse the differences among eight NRGs in 31 types of cancer based on all tumour tissues obtained from TCGA, their corresponding normal tissues and all transcriptome gene expression levels obtained by GTEx in different normal tissues. Two tumour types, mesothelioma (MESO) and uveal melanoma (UVM), were excluded from the analysis because of a lack of para‐cancerous tissue data. The related heatmap was drawn using the R package ‘ggplot2’. The darker the colour, the greater the difference in expression between tumour and normal tissues. The association of the eight NRGs in all tumours was analysed and visualized using the above method. The darker the colour, the stronger the association between two genes. The STRING database (https://string‐db.org/), an online database for building protein‐related interaction networks, was used and the interactions between eight NRGs and their proteins were obtained.

### 2.4 NRG survival analysis

The related clinical information from the TCGA database and the eight corresponding NRG expression levels were used to study the relationship between NRG expression and tumour prognosis via univariate cox analysis. Relative *p* values and hazard ratios were extracted, and a relative heatmap was drawn using the R package ‘pheatmap’. Genes marked in red and blue were considered risk and protective factors in a tumour, respectively (*p* < 0.05). Kaplan–Meier survival curves of NRGs in different tumours were then obtained using the GEPIA2 database (http://gepia2.cancer‐pku.cn/#index).

### 2.5 NRG mutation and methylation‐related analysis

Single nucleotide variation (SNV) is closely related to the tumour immune microenvironment and may be used as a prognostic marker of immune checkpoint inhibitors.^16^ A copy number variation (CNV) tends to make patients more susceptible to tumours.^17^, ^18^ Eight NRG‐associated mutations in tumours were explored using the GSCAlite database (http://bioinfo.life.hust.edu.cn/GSCA/#/). In the ‘Mutation’ module, all tumours were selected, and eight NRGs were inputted in turn. The SNV, CNV and methylation‐related status of these eight NRGs were also obtained in turn.

### 2.6 Determining necroptosis scores

According to the expression of eight NRGs in 10,497 samples, ‘single‐sample gene set enrichment analysis’ was conducted to score each sample for necroptosis. Then, the necroptosis scores of 33 types of cancer were arranged from small to large and were visualized as boxplots by using ggplot2. Differences in necroptosis scores between cancer and para‐cancerous tissues of specific cancer were analysed and visualized using the R package ‘ggpubr’.

### 2.7 Necroptosis scores and prognosis analysis

According to the necrotic apoptotic scores of different samples and related clinical information, correlation with prognosis was explored in terms of four indices: overall survival (OS), disease‐specific survival (DSS), disease‐free interval (DFI) and progression‐free interval (PFI). Univariate cox analysis was performed in R package ‘survival’ and ‘survminer’, and results were visualized with the R package ‘forestplot’. Then, the corresponding graph was drawn according to the *p* value.

### 2.8 Necroptosis gene set variation analysis (GSVA)

GSVA^19^ was conducted to further explore the possible enriched pathways associated with necroptosis. A class of hallmark gene sets from the MSigDB database, which included 50 enriched pathways closely related to tumours, was used. The correlation between necroptotic scores and related pathways in each sample was determined through ‘single‐sample gene set enrichment analysis’ (ssGSEA) and visualized with the R package ‘pheatmap’. Red and blue represented positive and negative correlation, respectively; the darker the colour, the stronger the correlation. The number of ‘*’ corresponded to the magnitude of the *p* value.

### 2.9 Necroptosis and tumour microenvironment analysis

Based on the ssGSEA, Estimation of STromal and Immune cells in MAlignant Tumour tissues using Expression data (ESTIMATE) was used to score the matrix of all tumours and the two gene sets of immunity. The relative tumour purity was obtained. Subsequently, the possible pathways of necroptosis and related tumour microenvironments were explored. The gene set of Mariathasan and colleagues^20^ were used: immune‐related pathways, Stroma/transfer‐related pathways and DNA damage repair‐related pathways. It was then visualized using ggplot2. Red and blue represented positive and negative correlation, respectively; the darker the colour, the stronger the correlation. The number of ‘*’ was equivalent to the magnitude of the *p‐*value.

### 2.10 Necroptosis and immunologic infiltration analysis

The relationship between necroptosis and invasion of fibroblasts and related immune cells was investigated based on the original data from the TIMER2 online database (version 2, http://timer.cistrome.org/) and the ImmuCellAI database (http://bioinfo.life.hust.edu.cn/ImmuCellAI#!/). Three algorithms (EPIC, MCPCOUNTER and XCELL) were used to analyse fibroblast infiltration, and six other algorithms (TIMER, EPIC, MCPCOUNTER, CIBORSOFT, CIBORSOFT‐ABS and QUAN) were used to examine immune cell infiltration. The results were then visualized using ggplot2.

The correlation between necroptosis and immune‐associated genes, including immune‐associated suppressor genes, immune‐associated activation genes in tumours, and genes related to chemokines, chemokine receptors, and major histocompatibility complex (MHC) was investigated. Visualization was performed using ggplot2.

In all visualizations, red and blue represented positive and negative correlation, respectively; the darker the colour, the stronger the correlation. The number of ‘*’ was equivalent to the magnitude of the *p*‐value of the correlation.

### 2.11 Necroptosis, TMB and MSI analysis

TMB is defined as the total number of somatic gene coding errors, base insertion, replacement or deletion errors detected per million bases.^21^ It is an important biological indicator of the degree of mutation in a tumour; that is, the higher the TMB, the better the outcome of tumour immunotherapy.^22^ MSI is a result of a defective DNA mismatch repair function in tumour tissues, and MSI‐H tumours often indicate better treatment outcomes.^23^ TMB data were obtained from the UCSC Xena database, and MSI data were collected from the research of Bonneville et al.^24^ Results were represented as a correlation radar graph by using the R package ‘FMSB’, where the number of ‘*’ corresponded to the *p*‐value.

### 2.12 Necroptosis and clinical treatment analysis

A group of patients with non‐small‐cell lung carcinoma and a group of patients with bladder carcinoma that received programmed death 1 immunotherapy were selected to investigate the prognostic value of necroptosis in immunotherapy. The patients were then divided into high and low groups according to their necroptosis scores. Kaplan–Meier survival curves and disease progression were obtained based on related clinical data.

According to the relevant data in the Genomics of Drug Sensitivity in Cancer (GDSC) database and the Cancer Therapeutics Response Portal (CTRP) database, the correlation between the eight NRGs and the sensitivity of tumour‐targeting therapy was studied. Red and blue indicated the positive and negative correlation between a gene and a drug, respectively.

### 2.13 Statistical analysis

Independent t and Spearman's tests were used to compare two groups of variables, a chi‐square test was conducted to analyse the clinical data and the Kaplan–Meier survival and logarithmic rank tests were conducted to examine single factor variables. All statistical analyses were performed using the R software (version 4.1.3), and statistical significance was set at *p* < 0.05.

## 3 RESULTS

### 3.1 Differences in NRG expression and correlation analysis

**FIGURE 1:**
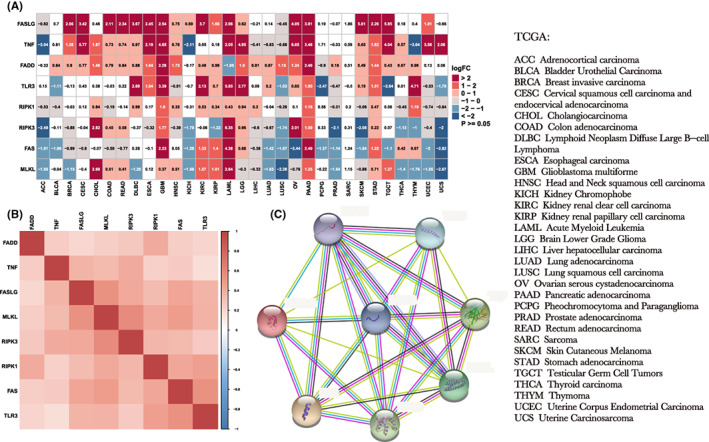
Expression of eight NRGs in different tumours and their correlations. The results are based on data collated from the TCGA and GTEx databases. (A) Analysis of differential expression of eight NRGs in tumour tissues and normal tissues. (B) Correlation analysis of eight NRGs. (C) Protein–protein interactions of eight NRGs. Red and blue represent positive and negative correlations, respectively, while white represents *p* > 0.05

To determine how different NRGs were expressed in each tumour, we analysed the expression of different genes in more than 10,000 samples from the TCGA database. Due to the lack of para‐cancerous tissue data for MESO and UVM, we selected 31 types of cancer tissues to compare with para‐cancerous tissue. As shown in Figure 1A, NRGs expression differed among the various tumours. In addition, the full name of the tumours in TCGA also be shown in Figure 1.

The subsequent correlation analysis of the eight NRGs showed that all NRGs were positively correlated with each other (Figure 1B). The eight NRG transcriptional proteins also interacted with each other (Figure 1C).

### 3.2 NRG prognostic analysis

**FIGURE 2:**
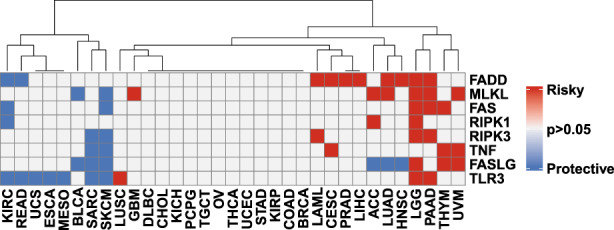
Relationship between the expression of eight NRGs and clinical prognosis in different tumours. Red and blue represent positive and negative correlations, respectively, while white represents *p* > 0.05

We performed univariate Cox analysis to determine whether NRGs were independent risk factors for 33 types of cancer based on the clinical data from the TCGA. As shown in Figure 2, each NRG played a unique role in different cancer types. For example, *FADD* is a risk factor, whereas *TLR3* is a protective factor for most tumours. We used the GEPIA2 database to obtain specific Kaplan–Meier survival curves (Figure S1).

### 3.3 NRG mutations and methylation analysis

**FIGURE 3:**
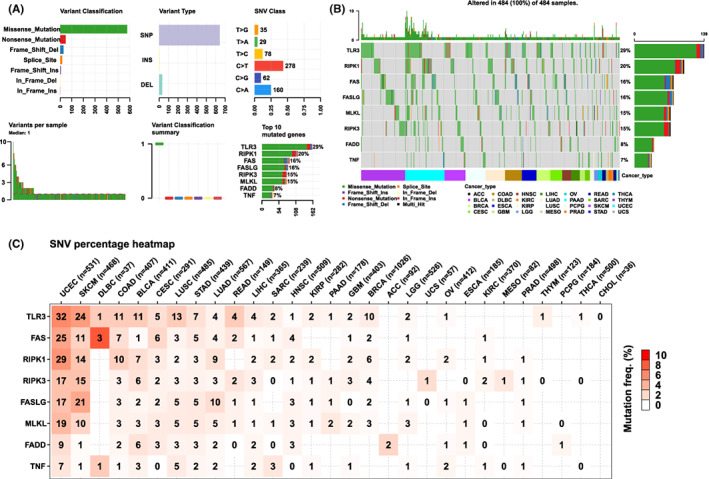
Mutation characteristics of eight NRGs in different tumours. The results are based on data collated from GSCAlite database. (A) Top left (variant classification): count of each type of deleterious mutation (missense mutation, nonsense mutation, frame shift ins, splice site, frame shift del, in frame del, in frame ins) of eight NRGs in different tumours. Top middle (variant type): count of the SNP and DEL of eight NRGs set in different tumours. Top right (SNV class): count of each SNV class of eight NRGs set in different tumours. Down left (variants per sample): count of variants in each sample. A bar indicates a sample, and the colour of the bar corresponds to the colour of the variant classification. Down middle (variant classification summary): the distribution of the count of each variant classification in the sample set of different tumours. The colour of the box corresponds to the colour of the variant classification. Down right (top 10 mutated genes): count and percentage of variants in the top 10 mutated genes. (B) Mutation distribution of the top 10 mutated genes from eight NRGs set in the sample set of selected cancers and the classification of SNV types. (C) Frequency of deleterious mutations in selected cancer types

We further explored the eight NRG mutations in tumours by using the GSCAlite database. We first analysed the SNV status of the NRGs. For example, Figure 3A,B show that the highest mutation rate of *TLR3* was 29%, followed by *RIPK1* with 20% and *TNF* with a low mutation rate of 7%. Most of the mutations were missense, followed by nonsense mutations. In Figure 3C, we explored SNV in all tumour samples; that is, SNV was found in 155 of 531 samples of uterine corpus endometrial carcinoma (UCEC), but in cholangiocarcinoma (CHOL), no SNV has occurred in any of the samples. Additionally, most samples were dominated by base C mutation, which was approximately four times as many as base T mutation.

**FIGURE 4:**
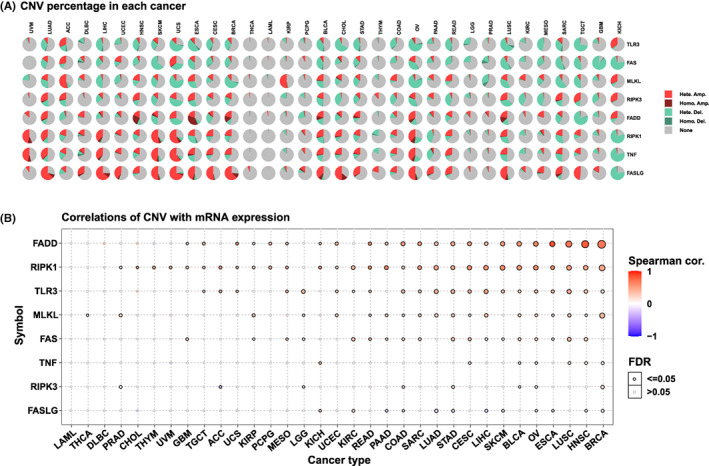
Mutation characteristics of eight NRGs in different tumours. The results are based on data collated from GSCAlite database. (A) CNV of eight NRGs in the selected cancer types. (B) Correlation of CNV with mRNA expression

We studied the CNVs of NRGs. For example, As shown in Figure 4A, the majority of CNVs in the eight NRGs were ‘Heterozygous Amplification’ (Hete.Amp) and ‘Heterozygous Deletion’ (Hete.Del), and the CNV of different genes in tumours varied. In particular, the CNV of *FASLG* was ‘Hete.Amp’. However, in kidney chromophobe (KICH), more than 75% of the samples showed ‘Hete.Del’. We also examined whether NRG expression was associated with CNV. CNV was positively correlated with the expression of NRGs except *FASLG*, *RIPK3* and *TNF* in most tumours (Figure 4B).

**FIGURE 5:**
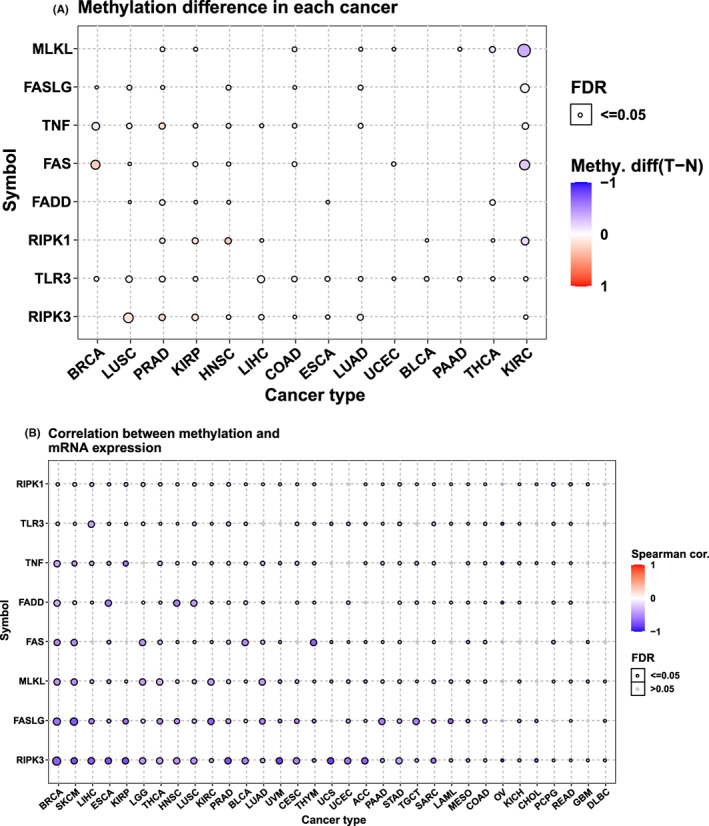
Relationship between eight NRGs and promoter methylation in different tumours. The results are based on data collated from GSCAlite database. (A) Methylation difference between tumour and normal samples of eight NRGs in selected cancers. (B) Correlation between methylation and mRNA expression

We examined the methylation status of eight NRGs. The expression of NRGs was significantly different from the methylation status only in 14 types of cancer tissues and normal tissues (Figure 5A). Interestingly, the NRG expression was negatively associated with methylation in almost all tumours (Figure 5B).

### 3.4 Necroptosis score

**FIGURE 6:**
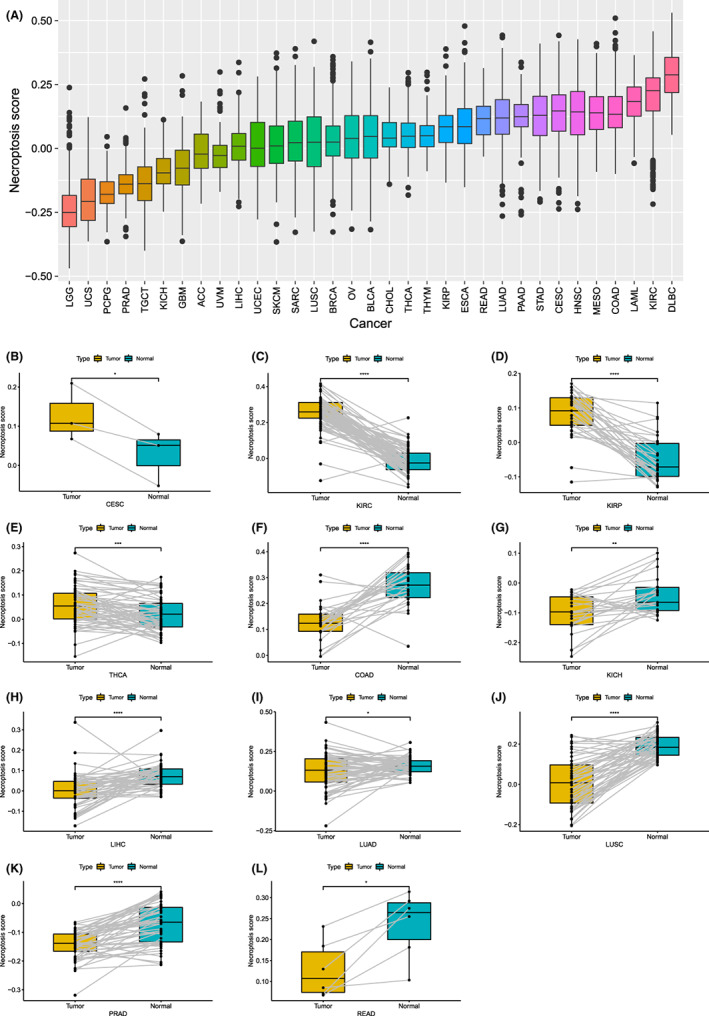
Necroptosis score of different tumours. (A) The size of the necroptosis score increased from left to right in each of the 33 tumours. (B) Differences in necroptosis scores between cancer and para‐cancerous tissues of KIRC, KIRP, THCA, COAD, KICH, LIHC, LUAD, LUSC and PRAD. *p < 0.05, **p < 0.01, ***p < 0.001, **** *p* < 0.0001

We performed ssGSEA to score each tumour sample for necroptosis (Table S1). We then ranked the scores of 33 types of cancer from low to high (Figure 6A). Brain lower‐grade glioma (LGG) and lymphoid neoplasm diffuse large B‐cell lymphoma had the lowest and highest necroptosis scores, respectively. In addition, we compared the necroptosis scores of the 31 types of cancer with those of their corresponding normal tissues and found significant differences among the 11 types of cancer (*p* < 0.05; Figures 6B–6L). The scores of cervical squamous cell carcinoma and endocervical adenocarcinoma, kidney renal clear cell carcinoma (KIRC), kidney renal papillary cell carcinoma (KIRP) and thyroid carcinoma (THCA) tumour tissues were higher than those of normal tissues were. Conversely, the scores of normal tissues were higher than those of colon adenocarcinoma (COAD), KICH, liver hepatocellular carcinoma (LIHC), lung adenocarcinoma (LUAD), lung squamous cell carcinoma (LUSC), prostate adenocarcinoma (PRAD) and rectal adenocarcinoma oesophageal carcinoma tumour tissues.

### 3.5 Necroptosis score and prognosis analysis

**FIGURE 7:**
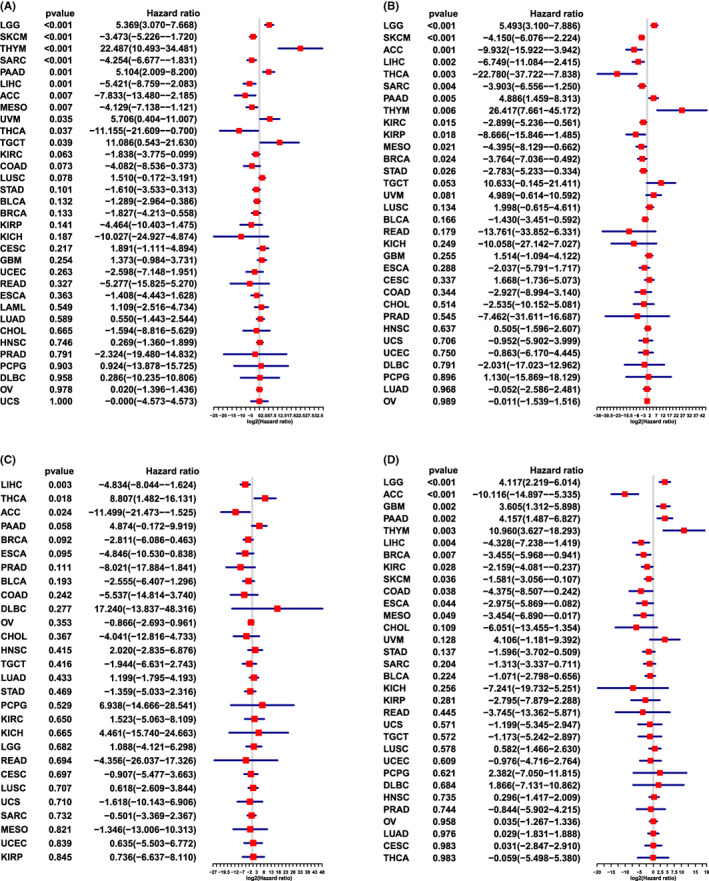
Relationship between necroptosis scores and OS, DSS, DFI and PFI in tumours. (A) Necroptosis score is associated with OS in 33 tumour types. (B) Necroptosis score is associated with DSS in 28 tumour types. (C) Necroptosis score is associated with DFI in 32 tumour types. (D) Necroptosis score is associated with PFI in 32 tumour types

We then performed a univariate cox analysis based on the sample's necroptosis score combined with the clinical data. We used OS (including 33 types of cancer), DSS (including 28 types of cancer), DFI (including 32 types of cancer, lacking acute myeloid leukaemia [LAML]), PFI (including 32 types of cancer, lacking LAML) and four indices to evaluate the correlation between necroptosis and prognosis and arrange them from low to high according to the *p*‐value. As shown in Figure 7A, the OS of 11 types of cancer was closely correlated with their necroptosis scores: LGG (*p* < 0.001), thymoma (THYM, *p* < 0.001), pancreatic adenocarcinoma (PAAD, *p* = 0.001), UVM (*p* = 0.035) and testicular germ cell tumours (TGCT, *p* = 0.039) were risk factors, and skin cutaneous melanoma (SKCM, *p* < 0.001), sarcoma (SARC, *p* < 0.001), LIHC (*p* = 0.001), adrenocortical carcinoma (ACC, *p* = 0.007), MESO (*p* = 0.007) and THCA (*p* = 0.037) were protective factors. As shown in Figure 7B, DSS in LGG (*p* < 0.001), PAAD (*p* = 0.005) and THYM (*p* = 0.006) were risk factors for necroptosis; SKCM (*p* < 0.001), ACC (*p* = 0.001), LIHC (*p* = 0.002), THCA (*p* = 0.003), SARC (*p* = 0.004), KIRC (*p* = 0.015), KIRP (*p* = 0.018), MESO (*p* = 0.021), breast invasive carcinoma (BRCA, *p* = 0.024) and stomach adenocarcinoma (STAD, *p* = 0.026) were protective factors for necroptosis. However, DFI was positively correlated with THCA (*p* = 0.018) and negatively correlated with LIHC (*p* = 0.003) and ACC (*p* = 0.024; Figure 7C). The correlation between PFI and necroptosis scores in the 12 types of cancer was statistically significant (Figure 7D) except for the following risk factors: LGG (*p* < 0.001), glioblastoma multiforme (*p* = 0.002), PAAD (*p* = 0.002), and THYM (*p* = 0.003), but in ACC (*p* < 0.001), LIHC (*p* = 0.004), BRCA (*p* = 0.007), KIRC (*p* = 0.028), SKCM (*p* = 0.036), COAD (*p* = 0.038), oesophageal carcinoma (ESCA, *p* = 0.044) and MESO (*p* = 0.049), which were protective factors.

### 3.6 Necroptosis GSVA

**FIGURE 8:**
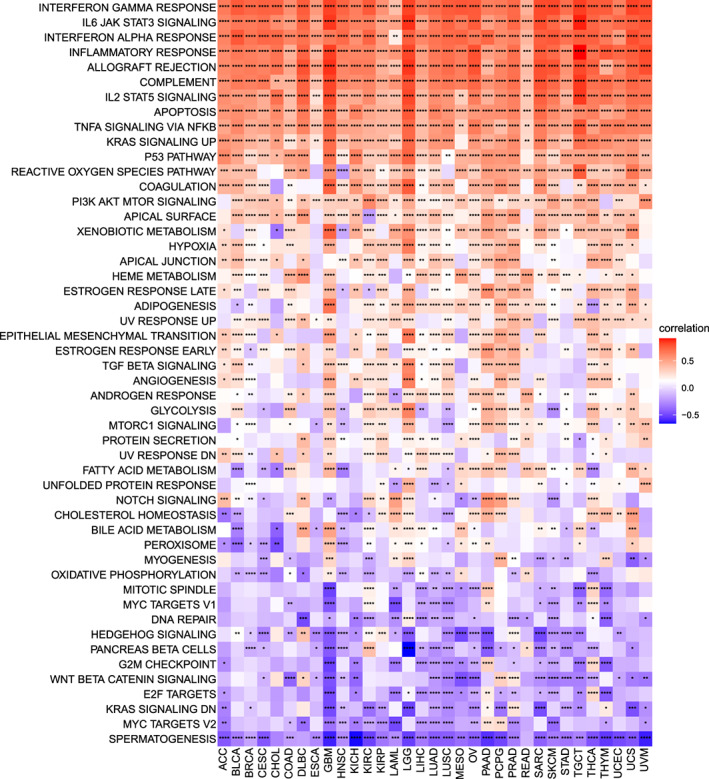
GSVA results. Results of HALLMARK enrichment analysis of necroptosis in different tumours. Red and blue represent positive and negative correlations, respectively, while white represents *p* > 0.05. *p < 0.05, **p < 0.01, ***p < 0.001, **** *p* < 0.0001

To investigate the possible signalling pathways of necroptosis in tumours, we used the hallmark gene set for the enrichment analysis of all tumours. Necroptosis was closely related to various pathways, especially interferon gamma response, interferon alpha response, IL6 JAK‐STAT3 signalling, complement, allograft rejection, inflammatory response, TNF‐α signalling via NFkb, apoptosis, IL2 STAT5 signalling, KRAS Signalling Up and p53 pathway (Figure 8); a positive correlation was observed in almost all cancer types.

### 3.7 Necroptosis and tumour microenvironment analysis

**FIGURE 9:**
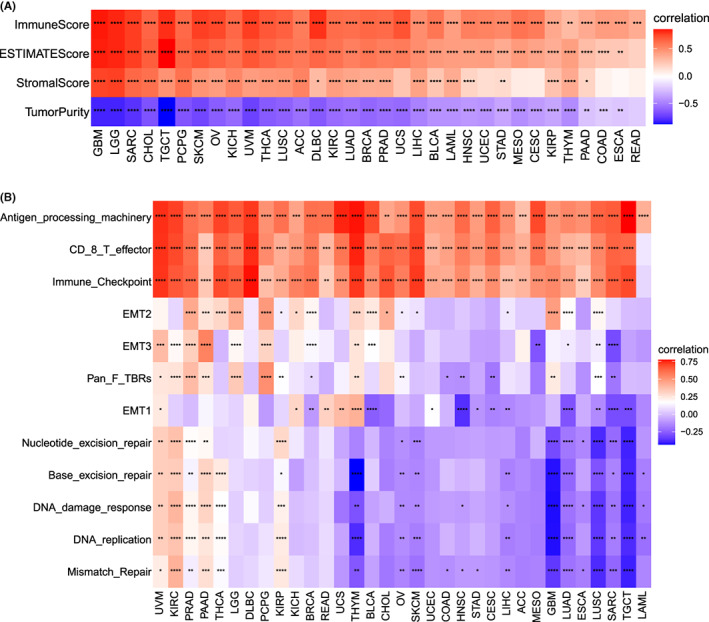
Relationship between necroptosis and the tumour microenvironment. (A) Correlation between necroptosis and stromal and immune scores. (B) Correlation between necroptosis and tumour microenvironment‐related pathways. Red and blue represent positive and negative correlations, respectively, while white represents *p* > 0.05. *p < 0.05, **p < 0.01, ***p < 0.001, **** *p* < 0.0001

We explored the relationship between necroptosis and the tumour microenvironment. Necroptosis was positively correlated with the immune microenvironment of all tumours and was closely related to the stromal microenvironment except for a few tumours (Figure 9A). We then investigated the possible signalling pathways of necroptosis in a tumour microenvironment (Figure 9B). Necroptosis was closely associated with antigen processing and immune checkpoints in all cancer types.

### 3.8 Necroptosis and immunologic infiltration analysis

**FIGURE 10:**
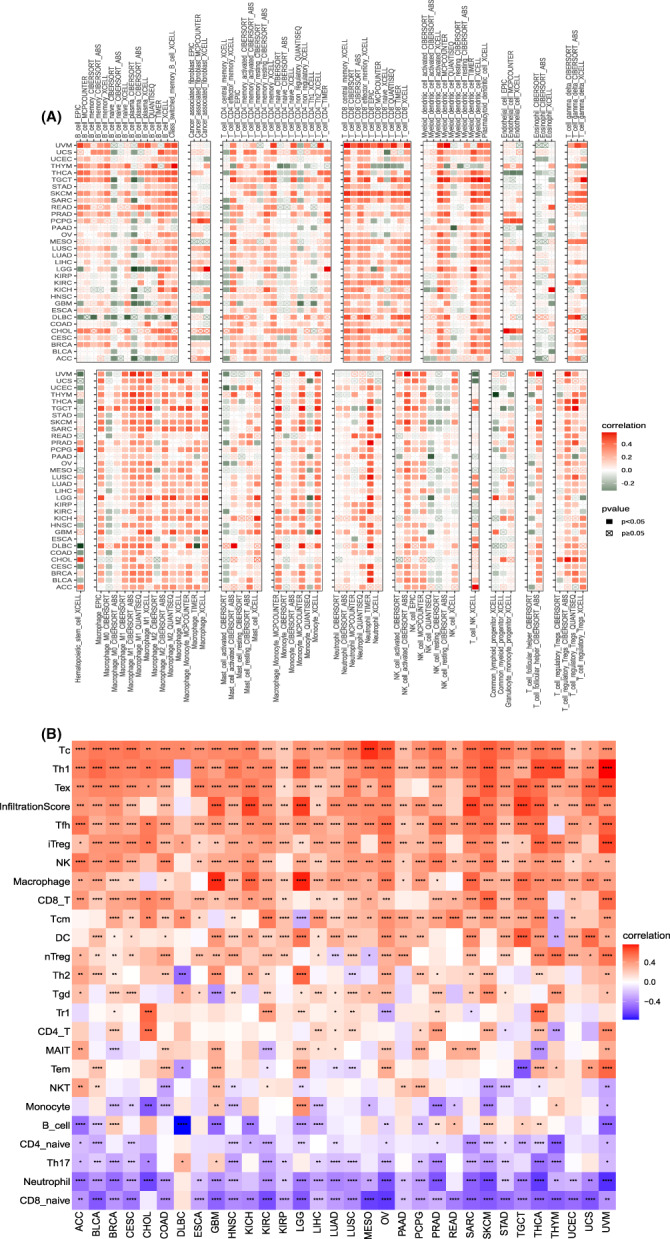
Relationship between necroptosis and tumour immune cell infiltration. (A) Relationship between necroptosis and tumour immune cell infiltration based on the TIMER2 database. (B) Relationship between necroptosis and tumour immune cell infiltration based on the ImmuneCellAI database. Red and blue or green represents positive and negative correlations, respectively, while white represents *p* > 0.05. *p < 0.05, **p < 0.01, ***p < 0.001, **** *p* < 0.0001

Based on the analysis of the role of necroptosis in a tumour microenvironment, we further examined the relationship between necroptosis and specific immune cell infiltration. Data from the TIMER2 database showed that CD8+ T cells, associated macrophages and neutrophils heavily infiltrated almost all cancer types when necroptosis was activated (Figure 10A). However, tumour‐associated fibroblasts were not associated with necroptosis, except in LGG, pheochromocytoma and paraganglioma (PCPG). We then continued to refine the analysis by using data from the ImmuneCellAI database (Figure 10B). Except for the infiltrating neutrophils in both databases with opposite results, the remaining infiltrating immune cells were essentially the same as the two results, indicating that tumour tissues contained a large number of infiltrating immune cells during necroptosis activation.

**FIGURE 11:**
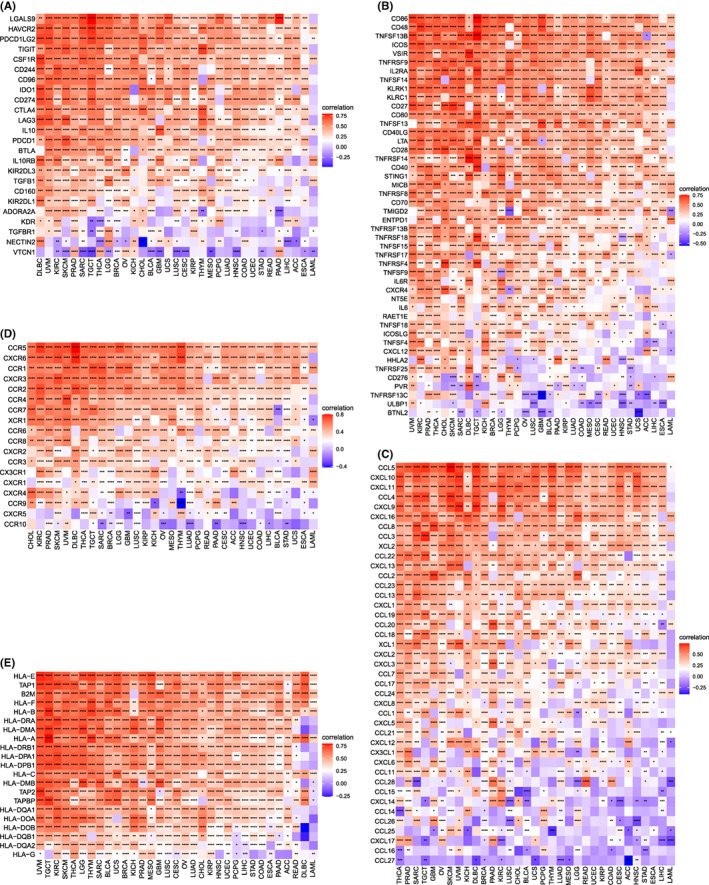
Relationship between necroptosis and immune‐associated genes. (A) Relationship between necroptosis and the immuno‐associated activating genes. (B) Relationship between necroptosis and the immuno‐associated suppressive genes. (C) Relationship between necroptosis and the genes‐related chemokine. (D) Relationship between necroptosis and the genes‐related chemokine receptor. (E) Relationship between necroptosis and the MHC genes. Red and blue represent positive and negative correlations, respectively, while white represents *p* > 0.05. *p < 0.05, **p < 0.01, ***p < 0.001, **** *p* < 0.0001

We then explored the correlation between necroptosis and immune‐related gene expression. Interestingly, immuno‐associated activating genes and immuno‐associated suppressive genes were positively associated with necroptosis in most cancer types (Figure 11A,B). For chemokines and chemokine receptors, specific genetic analyses were needed (Figure 11C,D), and the results varied among cancer types. Necroptosis was also positively correlated with MHC‐related genes (Figure 11E).

### 3.9 Necroptosis, TMB and MSI analysis

**FIGURE 12:**
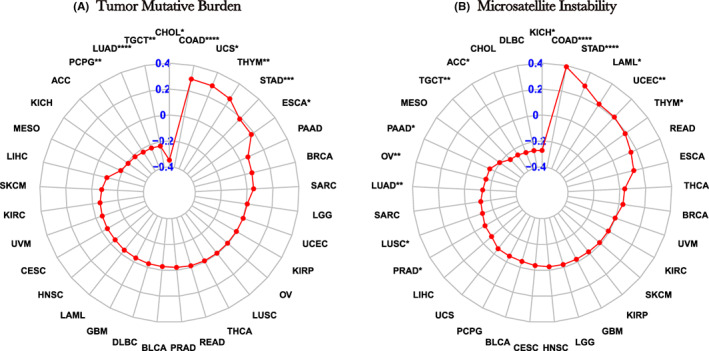
Relationship between necroptosis and TMB and MSI. (A) Radar plot of the correlation between necroptosis score and TMB in different tumours. (B) Radar plot of the correlation between necroptosis score and MSI in different tumours. *p < 0.05, **p < 0.01, ***p < 0.001, **** *p* < 0.0001

We also analysed the correlation of necroptosis with TMB and MSI (Figure 12A). The higher the score of necroptosis in COAD, uterine carcinosarcoma, THYM, STAD and ESCA, the higher the score of TMB; in PCPG, LUAD, TGCT and CHOL, on the other hand, the higher the score of necroptosis, the lower the score of TMB. MSI was positively correlated with necroptosis scores in COAD, STAD, LAML, UCEC and THYM (Figure 12B); however, negative correlation was observed in most of the remaining cancer types, such as PRAD, LUSC, LUAD, ovarian serous cystadenocarcinoma (OV), PAAD, TGCT, ACC and KICH.

### 3.10 Necroptosis and treatment analysis

**FIGURE 13:**
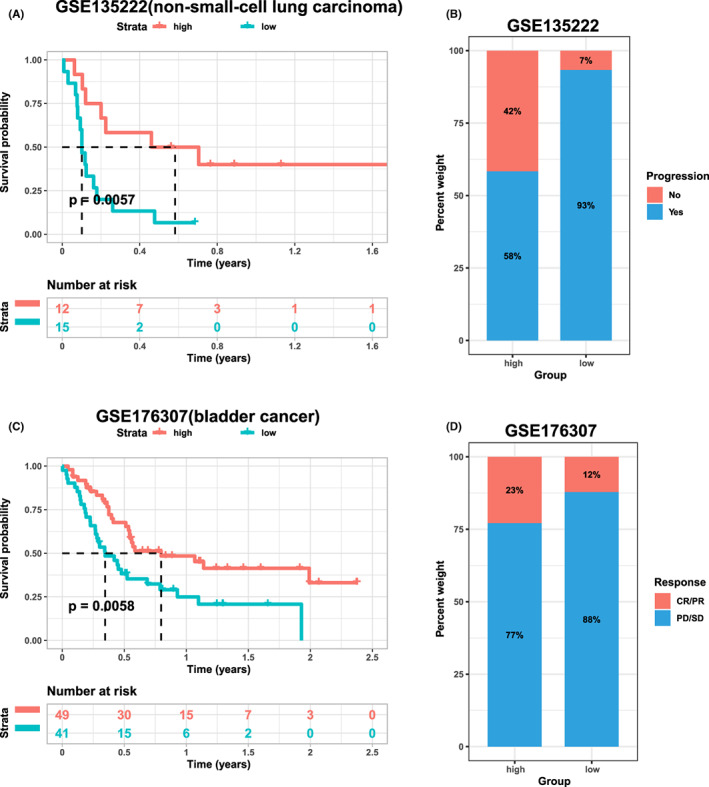
Relationship between the different necroptosis subgroups and immunotherapy prognosis. (A,B) Prognosis based on necroptosis score grouping of non‐small‐cell lung carcinoma patients that were treated with immunotherapy in GSE135222. (C,D) Prognosis based on necroptosis score grouping of bladder cancer patients that treated with immunotherapy in GSE176307

We selected two patient groups to explore the effect of necroptosis on the efficacy of immunotherapy. The higher the non‐small‐cell lung carcinoma score in GSE135222, the longer the survival events after immunotherapy (Figure 13A). The rate of tumour progression in patients with a high necroptosis score was significantly lower than that in patients with a low necroptosis score (Figure 13B). The higher the necroptosis score in GSE176307, the better the prognosis (Figure 13C,D).

We selected the GDSC and CTRP databases to investigate the sensitivity of the eight NRGs to 30 clinical targeted therapeutics. *FASLG* was not associated with most of the drugs (Figure S2). In the CTRP database, however, *TLR3*, *FADD* and *FAS* were positively associated with all drugs, whereas *TNF* and *RIPK3* were negatively associated with all drugs.

## 4 DISCUSSION

Necroptosis is a special cell death mechanism that affects cancer occurrence and development. However, the exact mechanisms that play similar or different roles in various cancer remain unknown. Pan‐cancer analysis of necroptosis has not been performed. Therefore, we used several databases to analyse the role of necroptosis in all cancer types.

First, we obtained eight genes closely related to necroptosis from the MSigDB database and analysed their gene expression, mutation, methylation and prognosis by using the TCGA, GTEX and GSCAlite databases. We found a reverse pattern of NRG expression even in the same type of cancer. The expression levels of *FASLG* and *FADD* in almost all cancer types were higher than those in normal tissues, and *FAS* was downregulated in most types of cancer. In pancreatic caricinoma, the eight NRGs were highly expressed in the tumour tissues to different degrees; combined with the follow‐up prognostic analysis, five of these NRGs were independent prognostic risk factors for pancreatic carcinoma, and this observation was consistent with previous findings.^15^, ^25^ Therefore, necroptosis likely plays a significant role in pancreatic carcinoma. Subsequent protein–protein interactions of NRGs also suggest that *RIPK1* and *RIPK3* are closely related to other NRGs as target genes of necroptosis.

In addition to differences in gene expression, mutations associated with NRGs in all types of cancer were studied through SNV and CNV. According to our results, the mutation rates of *TLR3* were 29% in all types of cancer and approximately 1% in patients with breast caricinoma. Susan et al.^26^ found that mutations in *TLR3* tend to reduce the BRCA risk, suggesting that *TLR3* may be a novel therapeutic target in breast carcinoma. Necroptosis also participates at early stages of UCEC.^27^ Our results also showed that the SNV mutation rate of NRGs in UCEC was 30%, and the tumour could be affected through this mechanism. With regard to CNV, a positive rate of approximately 40% was observed in all NRGs in breast carcinoma, which was similar to that in a previous study.^28^ However, the clinical significance of most mutations is uncertain, and further data analysis and sharing are needed to determine their effect on tumour susceptibility.

We then examined the methylation status of NRGs in cancer, with 14 types of cancer showing significantly different methylation levels from those of normal tissues. Most studies have shown that methylation occurs in a programmed manner,^29^ and necroptosis is a particular apoptotic process. Our results indicated that the methylation levels of NRGs in almost all types of cancer decreased as the NRG expression increase; however, further studies should determine whether a mechanism existed between them.

However, it is not enough to study necroptosis at the level of NRGs; therefore, we combined eight NRGs and evaluated all cancer samples for necroptosis. In previous studies related to NRG prognosis, the poor prognosis of tumours such as LGG is associated with a low necroptosis score, whereas the good prognosis of KIRC is related to a high tumour score. However, this is not absolute; SKCM and THYM scores are close to the gene expression level, and prognosis is completely different. Therefore, we explored the correlation between necroptosis score and prognosis and found that necroptosis was a risk factor for LGG, PAAD and THYM and a protective factor in SKCM and SARC. The results for PAAD and SARC were consistent with previous findings.^15^, ^25^, ^30^ Furthermore, necroptosis was present in the remaining types of cancer.

Subsequently, we discussed the possible mechanism of necroptosis in cancer and found that it is closely related to the immune response of tumours, in addition to some common pathways, such as p53, KRAS and JAK‐STAT3. We also investigated the relationship between necroptosis and the tumour microenvironment. Necrotic tissues release a large amount of damage‐associated molecular patterns (DAMPs) as a microenvironment, and DAMPs initiate an adaptive immune response.^31^ Necrotic tissues can provide dendritic cells with antigens and inflammatory cytokines and activate cytotoxic CD8+ T cells, thereby eliminating tumour cells.^14^ However, the release of DAMPs tends to promote inflammation, consequently promoting tumour angiogenesis, invasion and metastasis.^32^ This is consistent with our results, which showed that necroptosis and all types of cancer, except LAML, were associated with effector CD8+ T cells. We also found a correlation with immune checkpoints. Annelise et al.^33^ also observed that *RIPK1* and *RIPK3* activation might act in concert with immune checkpoints to affect tumours. Therefore, necroptosis is a valuable immunotherapy target. Based on specific immune cell infiltration, our results also suggested that a large number of immune cells infiltrate most types of cancer when necroptosis is activated. Thus, they have a potential benefit for immunotherapy.

To further elucidate the significance of necroptosis in immunotherapy, we studied the correlation between necroptosis and TMB and MSI. High TMB tends to indicate increased survival after immunotherapy,^34^ and our results revealed positive correlation between necroptosis score and TMB in most types of cancer. MSI‐H in patients with colorectal carcinoma benefits from immunotherapy with MSI.^35^ Our results also showed a positive association between MSI and necroptosis in gastrointestinal carcinoma, suggesting that necroptosis more likely benefits from immunotherapy when it is activated.

We validated our results with clinical data from two groups of cancer patients treated with immunotherapy. When the necroptotic score was relatively high, the patients survived longer than expected after immunotherapy, and the rate of cancer progression was even lower than usual. Interestingly, our results on the association between necroptosis and clinical targeted therapeutics indicated that *TLR3* and *FADD* tended to be more resistant to related drugs when they were expressed at high levels, whereas *TNF* showed the opposite trend. Thus, this study provided a new approach to the clinical treatment of cancer patients.

## 5 CONCLUSION

As a unique cell death process, necroptosis has been widely explored as a molecular mechanism that affects tumorigenesis and tumour development. In our study, eight NRGs and the necroptosis score of each type of cancer were used to explore their correlation. Despite relevant deficiencies, including a lack of relevant experimental validation, our results showed the correlation between necroptosis and cancer and the possible molecular mechanisms involved. Surprisingly, necroptosis has an important role in cancer immunity and is a potential new target of immunotherapy. Thus, it may serve as a basis for developing individualized therapy for clinical immunotherapy and has a broad research prospect.

## AUTHOR CONTRIBUTIONS

**Zheng Zhou:** Conceptualization (equal); methodology (equal); resources (equal); software (equal); writing – original draft (equal). **Jiajun Wu:** Conceptualization (supporting); project administration (supporting). **Wenli Ma:** Data curation (supporting); investigation (supporting). **Feilin Dong:** Funding acquisition (supporting); supervision (supporting). **Guowan Zheng:** Funding acquisition (supporting); supervision (supporting); writing – review and editing (supporting).

## FUNDING INFORMATION

This study was supported by the Medical and Health Science Research Fund of Zhejiang Province [2021KY055] and the Scientific Research Fund of Traditional Chinese Medicine of Zhejiang Province [2021ZA008].

## CONFLICT OF INTEREST

The authors declare that the research was conducted in the absence of any commercial or financial relationships that could be construed as a potential conflict of interest.

## Supporting information


Figure S1

Figure S2

Table S1

## Data Availability

The data used to support the findings of this study are available from the corresponding author upon reasonable request.
