# Supplementary figures and images for: Pan‐Cancer analyses of Necroptosis‐Related genes as a potential target to predict immunotherapeutic outcome
Source: J Cell Mol Med. 2022 Dec 29;27(2):204–21. doi: 10.1111/jcmm.17634 (PMC9843528; doi:10.1111/jcmm.17634)

# Figure S1

A

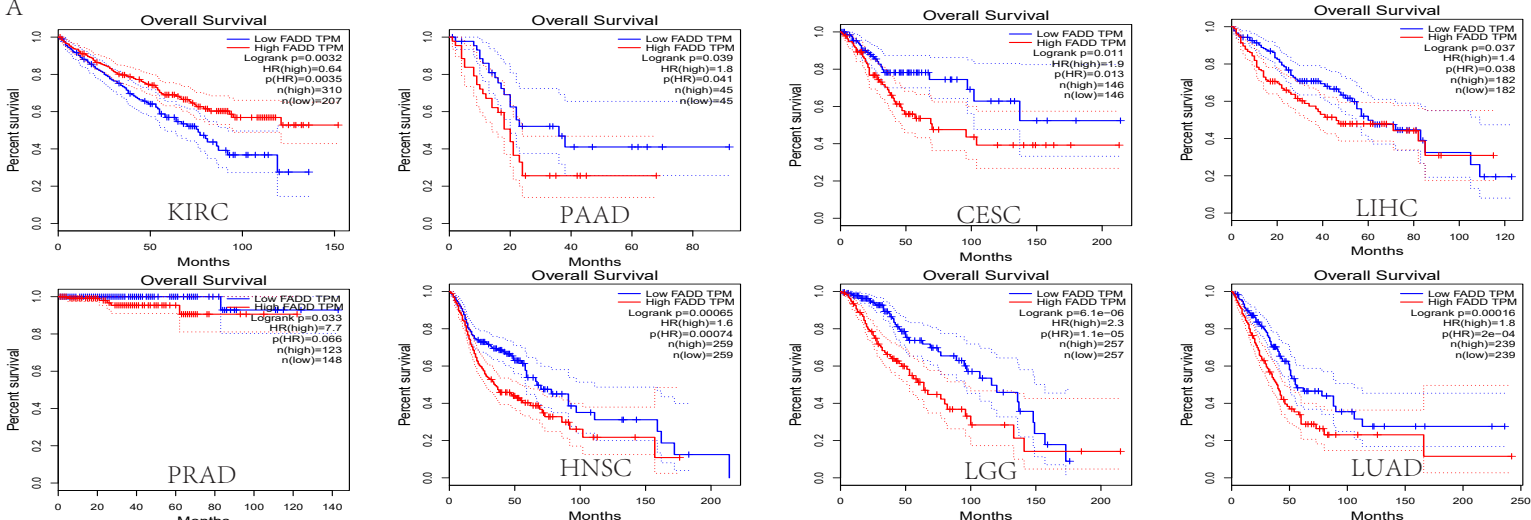

B

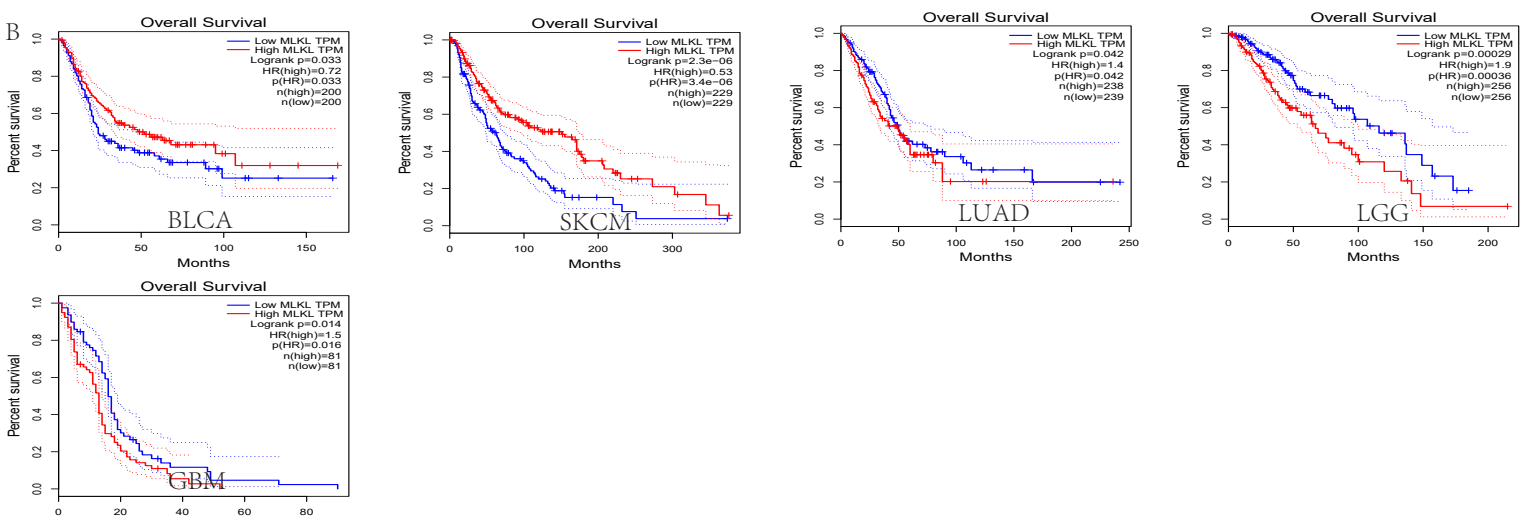

C

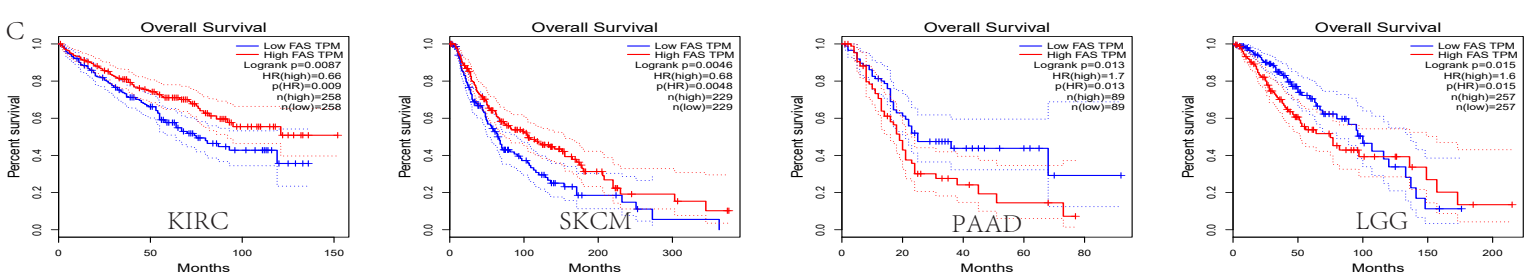

D

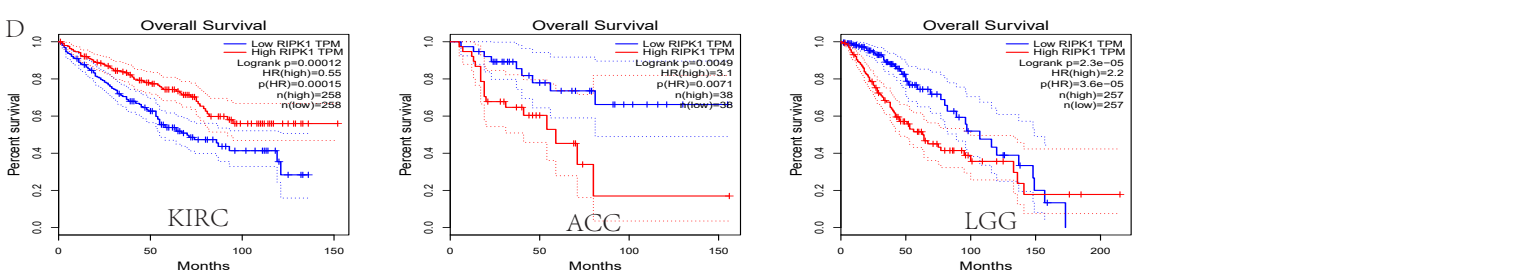

E

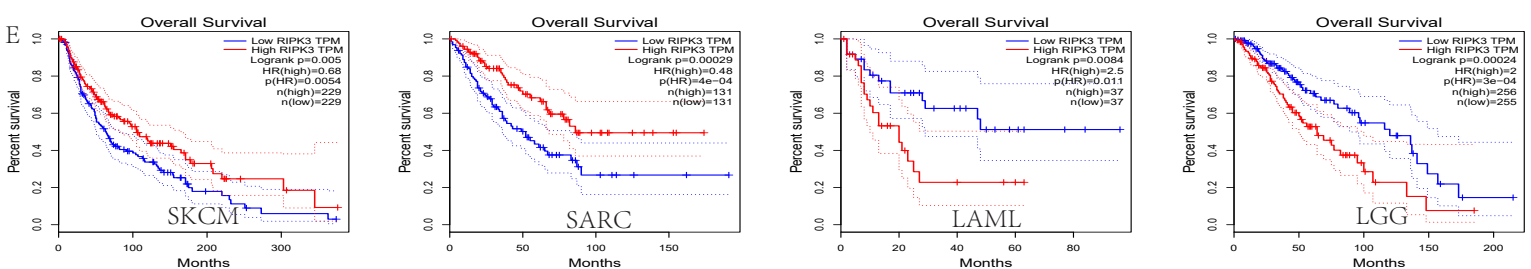

F

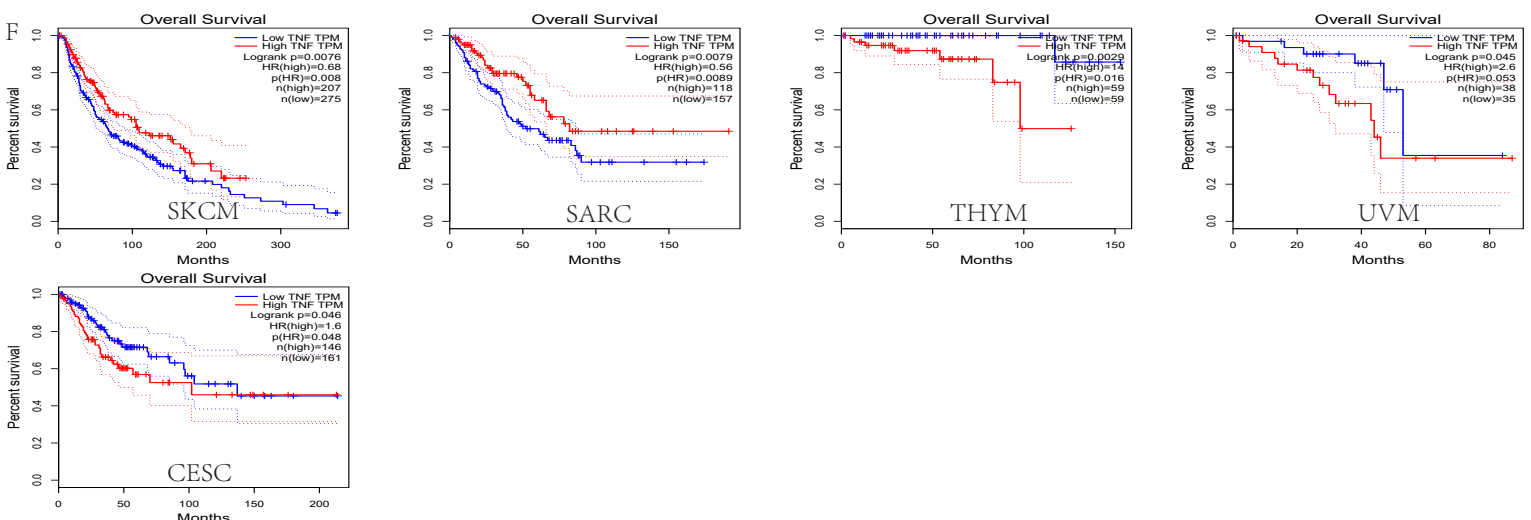

G

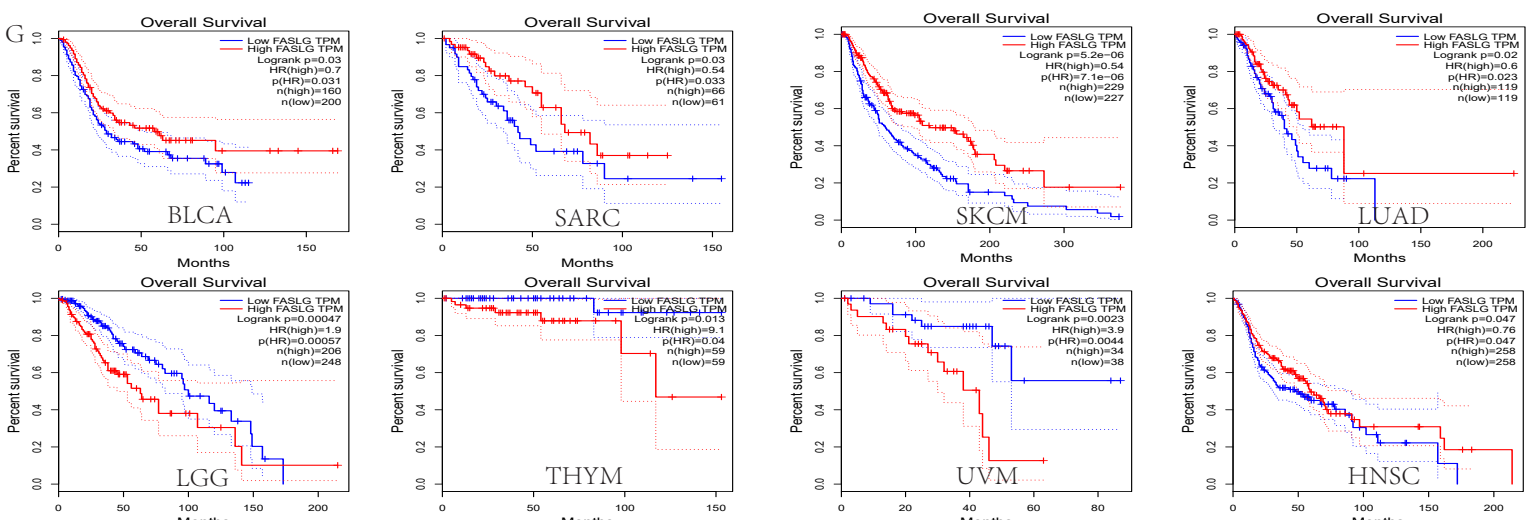

H

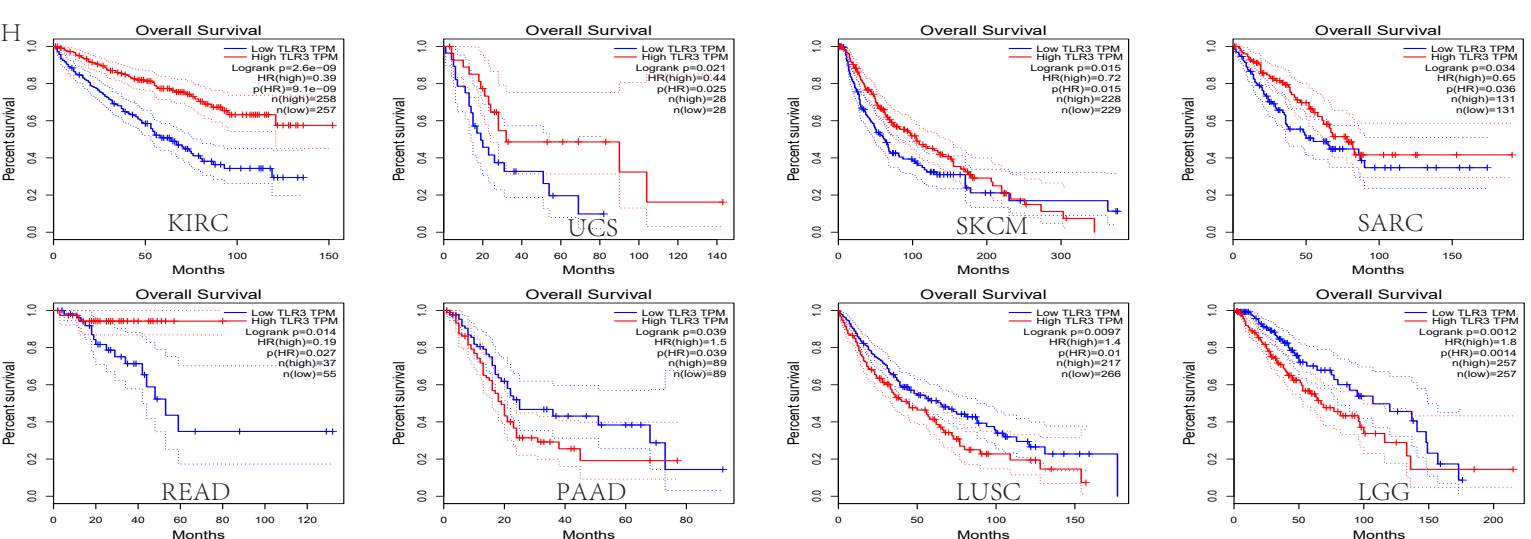

Supplement: Supplementary file 1 — Figure S1 [file JCMM-27-204-s002.pdf]

Figure S2

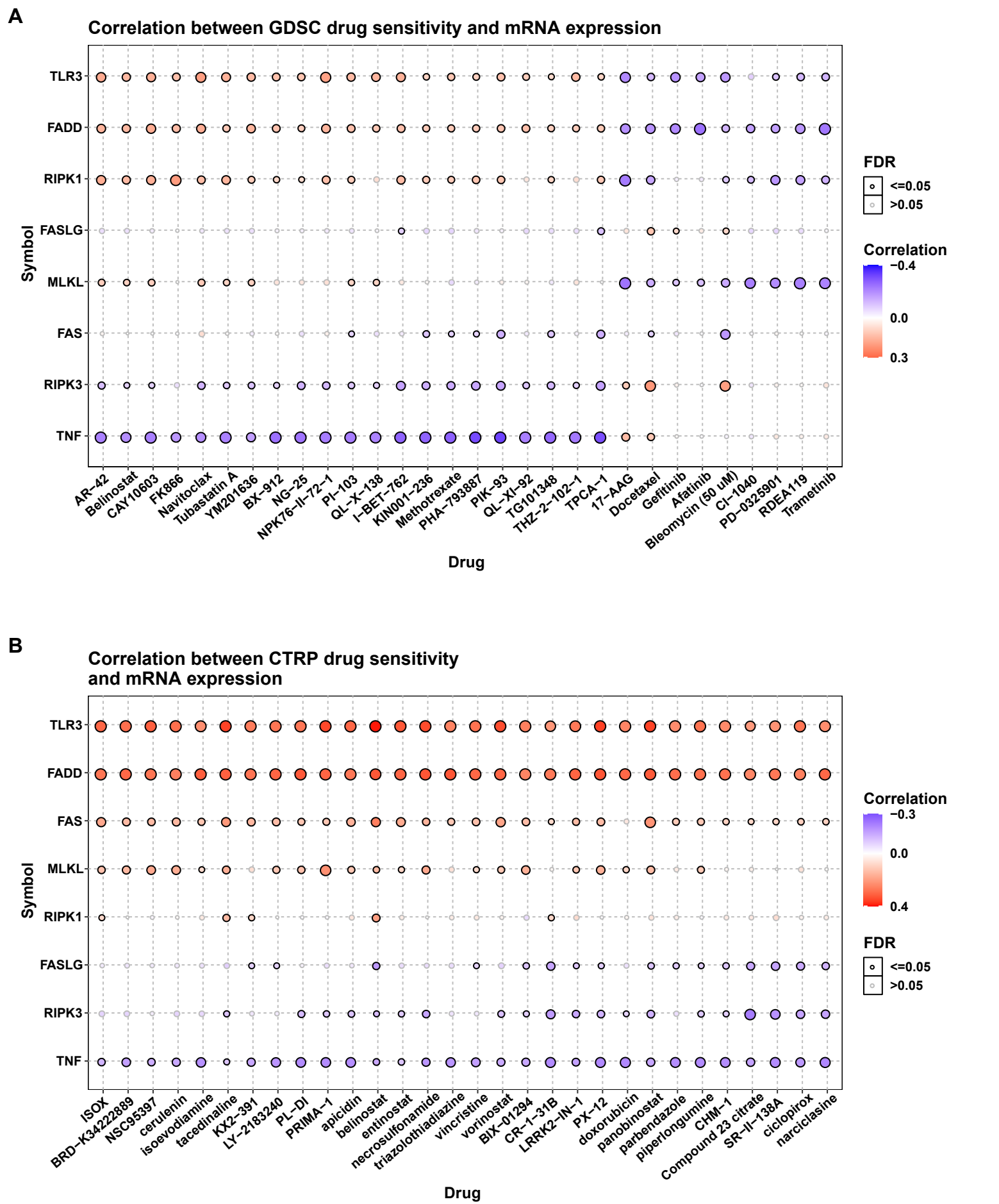

Supplement: Supplementary file 2 — Figure S2 [file JCMM-27-204-s003.pdf]
